# Dynamic Interaction Between Structural Asymmetry and Attention in the Right-Ear Advantage Revealed by MEG-Based ASSRs

**DOI:** 10.3390/brainsci16030286

**Published:** 2026-03-04

**Authors:** Keita Tanaka, Reo Yamada, Manami Kanamaru, Chie Obuchi, Hidehiko Okamoto, Takanori Kato, Hiromu Sakai

**Affiliations:** 1 School of Science and Engineering, Tokyo Denki University, Hiki-gun, Saitama 350-0394, Japan; 2 College of Engineering, Shibaura Institute of Technology, Koto-ku, Tokyo 135-8548, Japan; k-manami@shibaura-it.ac.jp; 3 Institute of Human Sciences, University of Tsukuba, Ibaraki, Tsukuba 305-8572, Japan; obuchi.chie.fu@u.tsukuba.ac.jp; 4 Department of Physiology, School of Medicine, International University of Health and Welfare, Narita, Chiba 286-8686, Japan; okamoto@iuhw.ac.jp; 5 Sumitomo Heavy Industries, Ltd., Shinagawa-ku, Tokyo 141-6025, Japan; takanori.kato@shi-g.com; 6 Faculty of Science and Engineering, Waseda University, Shinjuku-ku, Tokyo 169-8555, Japan; hsakai@waseda.jp

**Keywords:** dichotic listening test, right ear advantage, auditory steady-state response, magnetoencephalography, selective attention

## Abstract

**Background/Objectives**: The dichotic listening test (DLT) is widely used to assess auditory attention and hemispheric language lateralization, with the right-ear advantage (REA) representing a robust behavioral phenomenon. Although the REA is often attributed to structural asymmetries in auditory pathways and left-hemisphere dominance for speech processing, the neural mechanisms by which selective attention modulates this asymmetry remain unclear. This study examined how directed auditory attention influences the REA and its neural correlates using magnetoencephalography (MEG)-based auditory steady-state responses (ASSRs). **Methods**: Fifteen right-handed participants performed directed-attention dichotic listening tasks during MEG recording. One participant was excluded from MEG analyses due to excessive noise contamination, resulting in 14 participants included in neural analyses. Participants attended to either the left or right ear throughout each session and reported the perceived stimulus from the attended ear. Dichotic speech stimuli were amplitude-modulated at 35 Hz and 45 Hz for frequency tagging. ASSR amplitudes were extracted from the left and right auditory cortices and analyzed in relation to behavioral accuracy using correlation analyses and analysis of covariance (ANCOVA). **Results**: Behavioral accuracy was significantly higher during right-ear attention than left-ear attention, indicating a residual REA. ASSR amplitudes tended to be higher during left-ear attention. Importantly, during left-ear attention, ASSR amplitude in the left auditory cortex showed a significant positive correlation with behavioral accuracy, whereas no such association was observed during right-ear attention. **Conclusions**: These findings indicate that the REA reflects a dynamic interaction between structural auditory asymmetry and top-down attentional control, with successful left-ear listening relying on compensatory recruitment of the left auditory cortex.

## 1. Introduction

The dichotic listening test (DLT) has long served as a central paradigm for investigating auditory attention and hemispheric language lateralization. Early studies using dichotic listening demonstrated that verbal stimuli presented to the right ear are perceived more accurately than those presented to the left ear, a phenomenon later termed the right-ear advantage (REA) [1]. This seminal observation established the REA as a robust behavioral marker of hemispheric asymmetry in speech perception.

Subsequently, Kimura proposed a structural model to account for the REA, suggesting that auditory inputs are processed predominantly via contralateral pathways and that speech perception is dominated by the left hemisphere [2]. According to this model, right-ear stimuli gain more direct access to the language-dominant left hemisphere, whereas left-ear stimuli are initially processed in the right hemisphere and must undergo interhemispheric transfer via the corpus callosum. Together, these studies framed the REA because of fixed structural asymmetries within the auditory system.

Although this structural account successfully explains the baseline REA, subsequent behavioral studies have shown that auditory lateralization is not entirely fixed. In particular, manipulations of selective attention can substantially modulate ear advantages. Hugdahl and Andersson introduced the forced-attention paradigm, demonstrating that directing attention to the left ear reduces the REA, albeit without fully reversing it [3]. Building on this work, Hiscock and Kinsbourne argued that the REA reflects an interaction between attentional control mechanisms and inherent structural asymmetries rather than a purely anatomical phenomenon [4]. These findings suggest that top-down attentional processes can partially compensate for the structural disadvantages associated with left-ear input.

Neurophysiological studies further support the notion that selective attention dynamically modulates auditory cortical processing during dichotic listening. Functional and electrophysiological evidence indicates that attention enhances neural responses in auditory cortex and alters hemispheric balance depending on task demands. Alho et al. demonstrated that attention selectively modulates auditory cortical responses to speech sounds during dichotic listening, highlighting the role of cortical-level mechanisms in attentional control [5]. Using magnetoencephalography (MEG), Tanaka et al. reported that neural indices of auditory processing are sensitive to attentional manipulation and closely linked to behavioral ear advantages [6].

Auditory steady-state responses (ASSRs) provide a powerful tool for examining such attentional modulation of auditory cortical activity. ASSRs are phase-locked neural responses to periodic acoustic stimulation and are particularly robust in the gamma frequency range around 40 Hz [7,8]. Frequency-tagging approaches using ASSRs allow selective tracking of neural responses to concurrent auditory streams and have been widely applied to study auditory selective attention [9,10]. A recent systematic review further confirmed that gamma-band ASSRs are reliably modulated by attentional state and reflect functional engagement of auditory cortical networks [11].

Beyond basic auditory attention, alterations in ASSRs have been linked to clinical conditions involving impaired neural synchronization and cortical communication, such as schizophrenia, autism spectrum disorder, and auditory neuropathy [12,13,14,15]. These findings underscore the sensitivity of ASSRs to both functional and pathological variations in auditory cortical processing, highlighting their potential as objective neural markers.

While stimulus-driven factors such as lexical familiarity or semantic content have been reported to influence auditory cortical responses by modulating cognitive load, these effects alone cannot account for successful listening performance under conditions that require overcoming structural auditory asymmetries. Accordingly, the present study does not aim to dissociate semantic processing effects per se, but rather to clarify how selective auditory attention interacts with structural asymmetries of the auditory system to shape neural responses and behavioral performance during dichotic listening.

Specifically, we investigated whether the magnitude of auditory cortical engagement, as indexed by ASSR amplitude, predicts listening accuracy under different attentional conditions. We hypothesized that, during attention to the structurally disadvantaged left ear, successful performance would depend on compensatory recruitment of the language-dominant left auditory cortex. In contrast, during right-ear attention, performance was expected to rely primarily on automatic processing supported by structurally efficient pathways, resulting in a weaker association between neural response magnitude and behavioral accuracy. By combining a directed-attention dichotic listening task with MEG-based ASSR measurements, the present study aims to elucidate the dynamic neural mechanisms underlying the REA beyond fixed structural dominance.

## 2. Materials and Methods

### 2.1. Participants

Fifteen native Japanese speakers with normal hearing participated in the study (14 males, mean age 22.8 years, standard deviation (SD) = 0.87). One participant was excluded from MEG/ASSR analyses due to excessive noise contamination in the MEG recordings. Behavioral accuracy analyses included all 15 participants, whereas neural analyses were conducted on the remaining 14 participants. Handedness was assessed using the Japanese version of the Edinburgh Handedness Inventory, and all participants were classified as strongly right-handed. Participants were all undergraduate or graduate students at Japanese universities who were native speakers of Japanese. All procedures were performed in accordance with the Declaration of Helsinki and were approved by the Research Ethics Committee of Tokyo Denki University.

### 2.2. Stimuli and Experimental Design

We prepared two lists of 48 meaningful Japanese two-syllable words spoken in a female voice and created word pairs (namely, “/a/ /ka/” means “red” or “/ne/ /ko/” means “cat”) by sampling single items from both lists. The stimulus words were spoken for a duration of 330–503 ms. When applicable, a silent interval was added to create sound samples of a consistent length of 500 ms. We simultaneously presented stimulus sounds to the left and right ears at an inter-stimulus interval of 3 s at an intensity +50 dB SL.

To dissociate neural responses to the two simultaneously presented speech streams, we employed a frequency-tagging approach. In this method, each auditory stimulus stream is amplitude-modulated at a distinct modulation frequency, allowing the corresponding neural responses to be identified at those specific frequencies in the MEG signal. In the present study, stimuli presented to one ear were modulated at 35 Hz, whereas stimuli presented to the other ear were modulated at 45 Hz. These frequencies were selected within the gamma-band range, where auditory steady-state responses (ASSRs) are known to be robust and reliably measurable using MEG.

Because ASSRs oscillate at the same frequency as the stimulus modulation, neural activity evoked by each ear was isolated by applying narrow band-pass filters centered at 35 Hz (33–37 Hz) and 45 Hz (43–47 Hz), respectively. This procedure enabled separation of the tagged ASSR components in the time domain under dichotic listening conditions. The modulation depth was set to 100% to maximize steady-state entrainment. The assignment of modulation frequencies to left and right ears was fully counterbalanced across sessions to avoid systematic bias. Stimuli were presented dichotically, such that a different word was delivered simultaneously to each ear.

**Figure 1:**
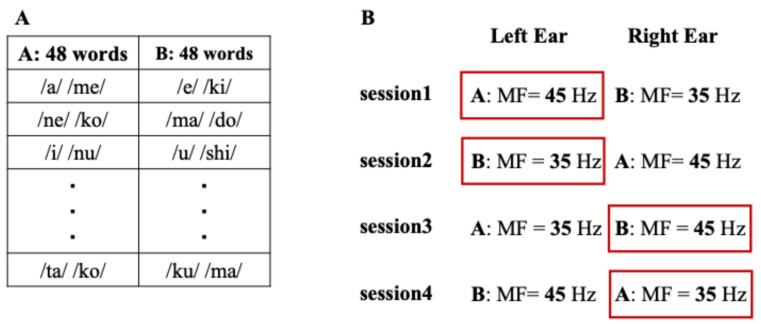
Experimental design and stimulus presentation. (**A**) Example of two stimulus sets (Set A and Set B) showing different presentation orders of the same 48 words for each participant, used to counterbalance order effects. (**B**) Schematic illustration of the presentation procedure in the dichotic listening task. The red frames indicate the ear to which attention was instructed prior to the task (attended ear). During each session, participants were instructed to continuously attend to either the left or right ear and to report only the word presented to the instructed ear throughout the entire session. MF denotes modulation frequency.

Stimulus timing was controlled using Presentation^®^ software (Version 23.1, Neurobehavioral Systems, Berkeley, CA, USA). Stimulus sounds were presented with insert earphones (Sensimetrics Model S14, Sensimetrics Co., Woburn, MA, USA). The experiment consisted of four sessions, during which participants were instructed to selectively attend to either the left or right ear and to continuously report only the word presented to the instructed ear throughout each session (Figure 1). The assignment of stimulus sets (Set A and Set B), presentation order, and modulation-frequency–ear pairing (35 Hz vs. 45 Hz) were fully counterbalanced across sessions and participants to avoid systematic bias. This design ensured that any observed effects could not be attributed to fixed stimulus order or frequency–ear assignment. Each session lasted approximately 45 min, including breaks to minimize fatigue.

### 2.3. MEG Data Acquisition

MEG data were recorded using a 64-sensor whole-head superconducting self-shielded MEG system (Sumitomo Heavy Industries Ltd., Yokosuka, Japan) located at Tokyo Denki University [16,17]. The MEG system was installed in a typical classroom in a university facility. A bus depot and an elevator were located approximately 10 m from the classroom, a highway approximately 1 km away, and a railroad line approximately 3 km away. This system overcomes the typical limitations of traditional MEG systems, such as the frequent need for liquid helium refills and the spatial constraints imposed by magnetically shielded rooms. The sampling rate was set to 1 kHz, and a notch filter at 50 Hz was applied to eliminate electrical noise.

**Figure 2:**
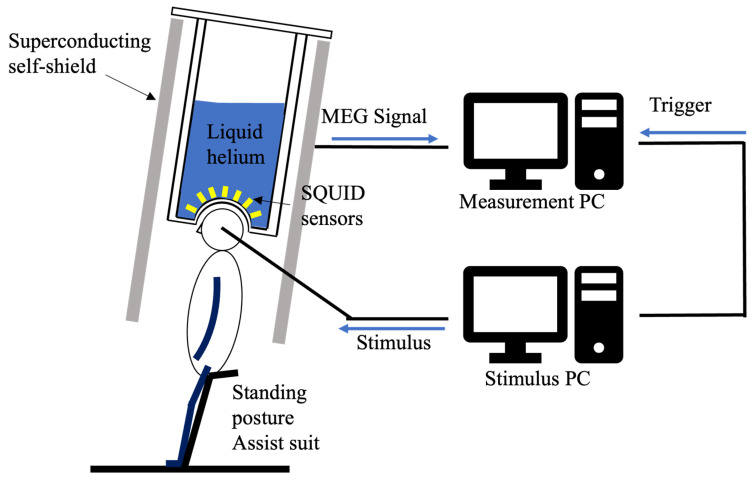
Schematic of the zero-boil-off cooling system for the superconducting magnetic shield and the MEG measurement environment. This setup illustrates the magnetic shield and equipment used for the auditory ASSR experiment.

Participants in the experiment were measured by MEG while standing. To suppress the generation of noise from the electromyography, they wore an assistance suit (archelis FX, Archelis Inc., Yokohama, Japan) to support their posture. To minimize movement artifacts, participants were instructed to keep their heads still during recording. Figure 2 shows the MEG measurement environment.

### 2.4. Data Analysis

MEG data preprocessing and analysis were conducted using MNE-Python (Version 1.9.0; open-source software developed by the MNE-Python community, USA; https://mne.tools, accessed on 1 February 2026). The steps included:

1. Epoching: Data were segmented into epochs from −500 ms to +1500 ms relative to stimulus onset.
2. Artifact Removal: Independent Component Analysis (ICA) was applied to remove ocular and muscle artifacts.
3. Averaging: Averaged waveforms of epochs with noise removed.
4. Combining: For the attended left-ear condition, averaged waveforms were computed by combining data from Session 1 and Session 2 for each sensor. For the attended right-ear condition, averaged waveforms were computed by combining data from Session 3 and Session 4 for each sensor.
5. Bandpass Filtering: The signals were filtered using bandpass filters at 33–37 Hz and 43–47 Hz to extract the ASSR components.
6. Baseline Correction: A pre-stimulus baseline of −500 to 0 ms was subtracted from each epoch.
7. Auditory Cortex Sensor Selection and ASSR Quantification: Sensors over bilateral temporal regions corresponding to auditory cortex, and signals from these sensors were averaged to obtain representative auditory cortical activity. The analytic signal was computed using the Hilbert transform, and ASSR amplitude was defined as the absolute value (envelope) of the complex-valued signal within the steady-state time window (500–1000 ms).
8. Statistical Analysis: Paired *t*-tests and ANOVA were performed to compare ASSR amplitudes and behavioral accuracy across conditions. Statistical significance was set at α = 0.05 (two-tailed) for all analyses.

To investigate the relationship between behavioral performance and neural activity, Pearson’s correlation coefficients were calculated between ASSR amplitudes and response accuracy for each participant. All ASSR analyses were conducted at the sensor level. Although no explicit source reconstruction was performed, previous MEG studies have consistently demonstrated that ASSRs in the gamma frequency range primarily originate from bilateral auditory cortices. Therefore, sensor-level ASSR measures were considered appropriate for assessing attentional modulation of auditory cortical activity in the present study.

## 3. Results

### 3.1. Behavioral Performance

**Figure 3:**
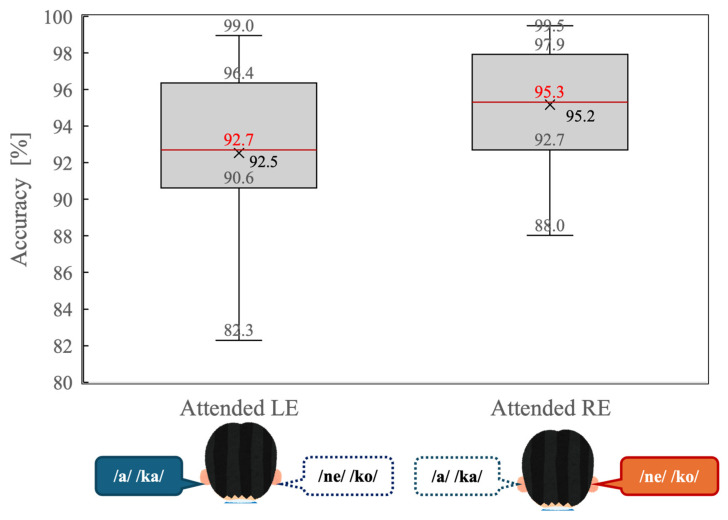
Distribution of behavioral accuracy (percentage of correct responses) during the dichotic listening task for the attended left-ear (Attended LE) and attended right-ear (Attended RE) conditions. Box plots indicate the median (red line), interquartile range (box), and minimum and maximum values excluding outliers (whiskers). “Xs” denote mean accuracy values.

Figure 3 illustrates behavioral accuracy for the dichotic listening task under the attended left-ear (Attended LE) and attended right-ear (Attended RE) conditions. Overall accuracy was high in both conditions, indicating that participants were able to perform the task reliably when attention was explicitly directed to one ear.

A paired-samples *t*-test revealed that accuracy was significantly higher in the Attended RE condition than in the Attended LE condition (t(14) = 2.34, *p* = 0.034). As shown in Figure 3, the median accuracy was greater during right-ear attention, and the interquartile range was slightly narrower compared with left-ear attention. These results indicate that a behavioral right-ear advantage persisted even under directed-attention conditions.

### 3.2. Auditory Steady-State Response (ASSR) Amplitudes

**Figure 4:**
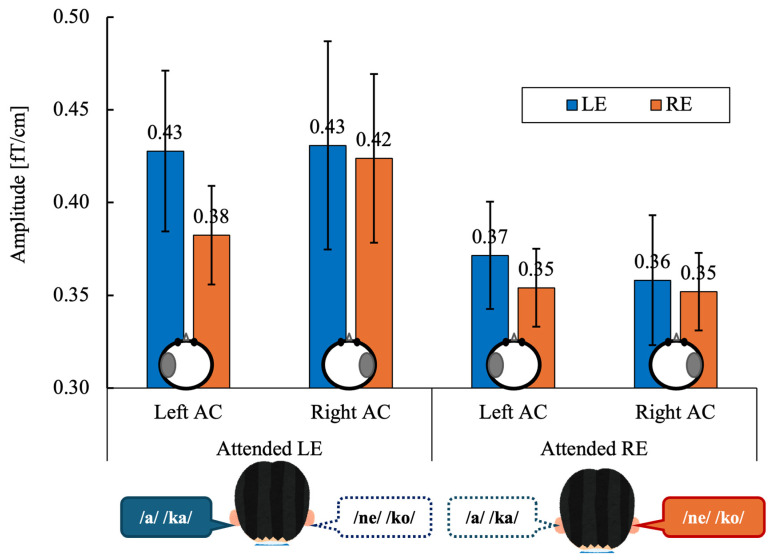
Grand-mean values across 14 participants of the ASSR amplitude in the left and right auditory cortex (Left AC or Right AC) to the left (LE) and right-ears (RE) during the attended left ear (Attended LE) and attended right ear (Attended RE) tasks. Error bars indicate standard error of the mean. The schematic circles shown below the bars indicate the hemisphere from which the ASSR amplitude was extracted (left or right auditory cortex). ASSR, auditory steady-state response; AC, auditory cortex, LE, left ear; RE, right ear.

Figure 4 shows the grand-mean ASSR amplitudes in the left and right auditory cortices (AC) evoked by left-ear (LE) and right-ear (RE) stimulation under directed-attention conditions. Data are shown separately for the attended left-ear and attended right-ear tasks.

ASSR amplitudes were averaged within each participant for each experimental condition. A three-way repeated-measures analysis of variance (ANOVA) was conducted with within-subject factors of Stimulated Ear (right vs. left), Attention (attend-right vs. attend-left), and Hemisphere (right vs. left). Statistical significance was set at α = 0.05 (two-tailed). When appropriate, effect sizes are reported as partial eta squared (ηp^2^). A three-way repeated-measures ANOVA revealed no significant main effect of Stimulated Ear (F(1, 13) = 0.51, *p* = 0.486, ηp^2^ = 0.038) or Hemisphere (F(1, 13) = 0.08, *p* = 0.775, ηp^2^ = 0.007).

The main effect of Attention did not reach statistical significance (F(1, 13) = 3.43, *p* = 0.087, ηp^2^ = 0.209), although ASSR amplitudes tended to be higher during left-ear attention than right-ear attention.

No significant two-way or three-way interactions were observed (all *p* ≥ 0.11; ηp^2^ range: 0.009–0.184).

### 3.3. Correlation Between ASSR and Behavioral Performance

**Figure 5:**
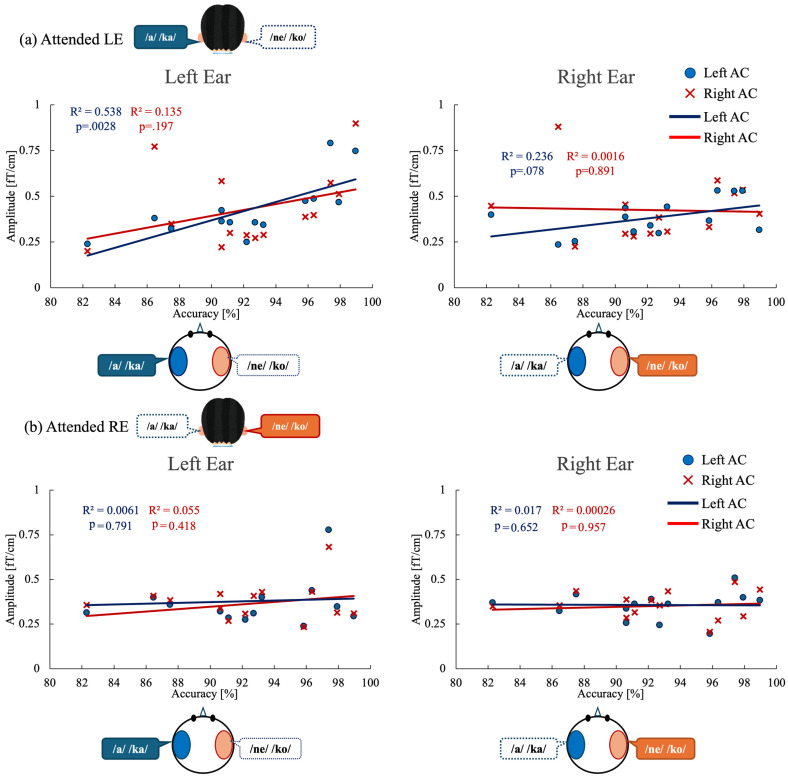
Correlations between behavioral performance and ASSR modulation in auditory cortex under each attention condition. (**a**) Attended left-ear condition. Left panel: Correlation between left-ear task accuracy and ASSR amplitude evoked by left-ear stimulation in the left auditory cortex (blue circles and regression line) and right auditory cortex (red crosses and regression line). Right panel: Correlation between left-ear task accuracy and ASSR amplitude evoked by right-ear stimulation in the left and right auditory cortices. (**b**) Attended right-ear condition. Left panel: Correlation between right-ear task accuracy and ASSR amplitude evoked by left-ear stimulation in the left and right auditory cortices. Right panel: Correlation between right-ear task accuracy and ASSR amplitude evoked by right-ear stimulation in the left and right auditory cortices. Each point represents an individual participant. R^2^ indicates the coefficient of determination for each linear regression fit. ASSR, auditory steady-state response; AC, auditory cortex.

To examine the relationship between behavioral performance and neural modulation, we analyzed correlations between task accuracy and ASSR amplitude in the auditory cortex (Figure 5). Linear regression analyses were conducted separately for the attended left-ear and attended right-ear conditions, focusing on ASSR amplitudes in the left and right auditory cortices.

For the attended left-ear condition (Figure 5a), a significant positive correlation was observed between ASSR amplitude in the left auditory cortex (Left AC) and left-ear stimulus accuracy (R^2^ = 0.538, *p* = 0.0028), indicating that greater neural engagement of the left auditory cortex was associated with better behavioral performance. In contrast, left-ear accuracy did not significantly correlate with ASSR amplitude in the right auditory cortex (Right AC) evoked by left-ear stimulation (R^2^ = 0.135, *p* = 0.197). Moreover, no significant correlations were found between left-ear accuracy and ASSR amplitudes evoked by right-ear stimulation in either the right auditory cortex (R^2^ = 0.0016, *p* = 0.89) or the left auditory cortex (R^2^ = 0.236, *p* = 0.078).

For the attended right-ear condition (Figure 5b), no significant correlations were observed between right-ear stimulus accuracy and ASSR amplitudes in either auditory cortex. Specifically, right-ear accuracy did not correlate with ASSR amplitude in the left auditory cortex evoked by left-ear stimulation (R^2^ = 0.0061, *p* = 0.791) or with ASSR amplitude in the right auditory cortex evoked by right-ear stimulation (R^2^ = 0.055, *p* = 0.418). Similarly, no significant relationships were found between right-ear accuracy and ASSR amplitudes evoked by right-ear stimulation in the left auditory cortex (R^2^ = 0.017, *p* = 0.652) or by left-ear stimulation in the right auditory cortex (R^2^ = 0.00026, *p* = 0.957).

To determine whether the observed ASSR–behavior relationship was independent of hemispheric effects, we further conducted an analysis of covariance (ANCOVA). For the attended left-ear condition, ANCOVA revealed a significant main effect of ASSR amplitude on behavioral accuracy (F(1, 24) = 9.73, *p* = 0.005), whereas neither the main effect of hemisphere (*p* = 0.176) nor the ASSR × hemisphere interaction (*p* = 0.143) reached statistical significance. In contrast, for the attended right-ear condition, ANCOVA revealed no significant effects of ASSR amplitude (*p* = 0.956), hemisphere (*p* = 0.727), or their interaction (*p* = 0.719) on behavioral accuracy. Consistent with these findings, the overall model for the attended right-ear condition was not significant (F(3, 24) = 0.07, *p* = 0.975), indicating that variability in ASSR amplitude did not account for performance during right-ear attention.

## 4. Discussion

The present study extends previous dichotic listening research by demonstrating that the behavioral relevance of auditory steady-state responses is condition-dependent, emerging specifically during left-ear attention when compensatory neural recruitment is required. The present study provides converging behavioral and neurophysiological evidence that the right-ear advantage (REA) in dichotic listening reflects a dynamic interaction between structural auditory asymmetry and top-down attentional control, rather than a fixed consequence of hemispheric dominance alone. By combining directed-attention dichotic listening with MEG-based auditory steady-state responses (ASSRs), we were able to dissociate automatic and compensatory neural mechanisms supporting speech perception under different attentional demands. A relevant precursor to the present study is our previous work [6], which employed a free-response dichotic listening paradigm. In that study, participants were not given explicit attentional instructions, and behavioral data indicated an automatic bias toward the right ear. Importantly, right-ear accuracy was positively correlated with ASSR amplitude in the left auditory cortex, suggesting that spontaneous right-ear dominance is supported by efficient left-hemispheric engagement. In contrast, the present study imposed explicit selective attention to one ear at a time. This distinction is theoretically critical: whereas our earlier findings reflected automatic attentional bias under free listening, the current paradigm required intentional top-down modulation, particularly under left-ear conditions where structural asymmetry must be overcome. The present results therefore extend our previous work by demonstrating that compensatory neural recruitment becomes behaviorally relevant when top-down control counteracts inherent structural bias.

At the behavioral level, participants demonstrated significantly higher accuracy during the attended right-ear condition than during the attended left-ear condition (Figure 3), indicating that a residual REA persisted even when attention was explicitly directed. This finding is consistent with classical accounts of dichotic listening, which attribute the REA to the more direct contralateral projection from the right ear to the language-dominant left hemisphere [2]. Importantly, however, the magnitude of this asymmetry was reduced relative to undirected listening reported in prior studies, suggesting that top-down attention partially mitigates—but does not abolish—the inherent structural bias favoring right-ear input. This behavioral pattern aligns with previous works showing that attentional instructions can attenuate ear advantages by reallocating processing resources toward the instructed ear [3,4].

Neurophysiological results further clarify how attentional modulation interacts with auditory lateralization. Analysis of mean ASSR amplitudes did not reveal a statistically significant main effect of attention, although a numerical trend toward larger responses during left-ear attention was observed (Figure 4). This global enhancement suggests that attending to the left ear imposes greater cognitive and neural demands, consistent with the notion that processing left-ear speech requires additional resources due to its indirect access to the left-hemisphere language network. Notably, no significant hemisphere × attention interaction was observed at the level of mean ASSR amplitude, indicating that attentional enhancement was not confined to a single auditory cortex. This finding argues against a simplistic contralateral-only model of attentional gain and instead supports a more distributed cortical engagement during effortful listening.

Crucially, correlation analyses revealed a condition-specific coupling between neural activity and behavioral performance. During the attended left-ear condition, left-ear accuracy showed a robust positive correlation with ASSR amplitude in the left auditory cortex (Figure 5a), whereas no such relationship was observed with right auditory cortex responses or with ASSRs evoked by right-ear stimulation. In contrast, during the attended right-ear condition, no significant correlations were found between behavioral accuracy and ASSR amplitude in either auditory cortex, regardless of stimulation ear (Figure 5b). This dissociation indicates that variability in neural engagement becomes behaviorally relevant only under demanding listening conditions—in this case, when attention must be directed to the structurally disadvantaged left ear.

These findings support a compensatory recruitment framework in which the left auditory cortex plays a pivotal role in overcoming the limitations of left-ear input. Under right-ear attention, speech processing benefits from automatic, structurally efficient routing to the left hemisphere, rendering additional modulation of cortical responses less critical for performance. Consequently, ASSR variability in either auditory cortex does not predict behavioral outcomes in this condition. By contrast, under left-ear attention, successful perception depends on the extent to which left-hemisphere auditory regions are actively engaged, despite the initial processing of left-ear input in the right hemisphere. The strong association between left auditory cortex ASSR amplitude and left-ear accuracy thus reflects compensatory recruitment of the language-dominant hemisphere via interhemispheric transfer.

This interpretation is consistent with prior neuroimaging and electrophysiological studies demonstrating attentional modulation of auditory cortex activity during dichotic listening. Alho et al. [18] and Jäncke et al. [19] reported enhanced auditory cortical responses under focused-attention conditions, while Payne et al. [20] showed that attentional bias toward the right ear is reflected in lateralized oscillatory dynamics. More recently, Elyamany et al. [21] demonstrated that directing attention to the left ear increases interhemispheric connectivity between auditory cortices, supporting the notion that compensatory cross-hemispheric interactions facilitate left-ear processing. Our results extend this literature by showing that such compensatory mechanisms are not merely present at the group level, but directly linked to individual differences in behavioral performance.

The absence of a significant ASSR × hemisphere interaction in the ANCOVA suggests that compensatory recruitment is not reflected in overall hemispheric dominance, but rather in the behavioral relevance of left auditory cortex activity under demanding conditions. Rather than indicating hemispheric equivalence, this result suggests that both auditory cortices contribute to listening performance, but that the behavioral relevance of their activity depends on task demands. Specifically, left auditory cortex engagement emerges as a limiting factor only when structural constraints necessitate compensatory processing, as in left-ear attention. This nuanced pattern reconciles classical structural models of the REA [1,2] with contemporary views emphasizing attentional control and network flexibility.

Despite the strengths of the present study, several limitations should be acknowledged. First, hemispheric language dominance was inferred from handedness rather than directly assessed using functional measures. Although all participants were strongly right-handed, and right-handedness is highly associated with left-hemisphere language dominance, individual variability cannot be excluded. The inclusion of participants with atypical or bilateral language lateralization could potentially influence the observed neural–behavioral relationships. Future studies incorporating direct measures of language lateralization, such as functional neuroimaging or dichotic consonant–vowel paradigms, would help clarify the extent to which the present findings depend on hemispheric dominance.

Second, although the present design enabled direct comparison between left- and right-ear selective attention, no explicit passive listening condition was included. Therefore, while the observed differences are most plausibly interpreted as attention-related modulation, the contribution of purely stimulus-driven neural entrainment cannot be entirely excluded. Future studies incorporating passive listening controls would further clarify the relative contributions of stimulus-driven and attention-dependent components.

Third, although frequency tagging allows dissociation of concurrent auditory streams, amplitude-modulated stimuli may impose cognitive demands that differ from natural speech perception, potentially influencing neural responses independently of attentional allocation. Fourth, while 100% amplitude modulation enhanced ASSR detectability, it may have altered the speech envelope and reduced transient consonant cues. Future studies incorporating passive controls, untagged stimuli, systematic assessment of language lateralization, and systematically varying modulation depth will be necessary to clarify the generalizability and boundary conditions of the compensatory mechanisms observed here.

In summary, the present findings demonstrate that the REA is best understood as a context-dependent phenomenon arising from the interplay of automatic processing and compensatory neural recruitment. Right-ear attention relies predominantly on efficient, structurally supported pathways, whereas left-ear attention engages adaptive mechanisms that recruit the left auditory cortex to support accurate perception. By highlighting the condition-specific relationship between ASSR modulation and behavioral performance, this study underscores the value of MEG-based ASSRs as objective markers of auditory attention and compensatory processing. Such measures may prove useful for assessing individual differences in listening strategies and for developing interventions aimed at improving auditory perception under challenging conditions.

## 5. Conclusions

This study investigated the neural mechanisms underlying the right-ear advantage (REA) in dichotic listening by combining directed-attention tasks with MEG-based auditory steady-state responses (ASSRs). Behavioral results demonstrated that accuracy remained significantly higher during right-ear attention than left-ear attention, indicating a residual REA even under explicit attentional control. Neurophysiological analyses revealed that, while ASSR amplitudes showed a numerical increase during left-ear attention, only left auditory cortex ASSR activity was positively associated with behavioral performance. No such association was observed during right-ear attention. These findings indicate that right-ear listening relies primarily on automatic, structurally supported processing, whereas successful left-ear listening requires compensatory recruitment of the language-dominant left auditory cortex. Together, the results redefine the REA as a dynamic, context-dependent phenomenon arising from the interaction between structural asymmetry and top-down attentional modulation. ASSRs may thus serve as objective markers of compensatory auditory processing capacity under challenging listening conditions.

## Data Availability

The data presented in this study are not publicly available due to ethical and privacy restrictions related to human participant data but are available from the corresponding author upon reasonable request.

## Abbreviations

The following abbreviations are used in this manuscript:

ASSR	Auditory Steady-State Response
DLT	Dichotic Listening Test
ICA	Independent Component Analysis
MEG	Magnetoencephalography
REA	Right Ear Advantage
SD	Standard Deviation
SE	Standard Error
